# Improvement mechanism of cellulose nanocrystal in coordination with calcium ions on the thermal gelation of soybean protein amyloid fibrils

**DOI:** 10.1016/j.fochx.2025.103275

**Published:** 2025-11-07

**Authors:** Shanlong Zhu, Xinyuan Song, Kang Zhong, Lu Lin, Ye Huang, Wenbin Zha, Yingnan Liu, Wei Lan, Yaqing Xiao

**Affiliations:** a Anhui Ecological Fermentation Engineering Research Center for Functional Fruit Beverage, Fuyang Normal University, Fuyang 236037, China; b Key Laboratory of Jianghuai Agricultural Product Fine Processing and Resource Utilization of Ministry of Agriculture and Rural Affairs, College of Food and Nutrition, Anhui Agricultural University, Hefei 230036, China

**Keywords:** Protein amyloid fibril, Cellulose nanocrystal, CaCl_2_, Composite gel, Synergistic effect

## Abstract

This study investigated the mechanism by which cellulose nanocrystals (CNC) derived from pear peel pomace synergize with Ca^2+^ to regulate the thermal gelation of soy protein amyloid fibrils (SAFs). Results showed that the combined use of 0.09 % (*w*/*v*) CNC and 400 mmol Ca^2+^ significantly enhanced SAFs gel strength and viscoelasticity, promoting the formation of β-sheet conformation and exposure of aromatic amino acid residues in SAFs. CNC reinforced hydrogen bonds to form excellent water-holding gel units, whereas Ca^2+^ strengthened ionic bonds and hydrophobic interactions to construct low water-holding gels with high strength. Mechanistic analysis revealed that CNC binds to specific SAFs amino acid sites via van der Waals forces and hydrogen bonds, while Ca^2+^ enhances CNC-SAFs crosslinking through electrostatic shielding effects. This study's new insights into the synergistic regulation of polysaccharide ions in SAFs gels could be applied to functional gel foods with controllable gel strength and WHC properties.

## Highlights

• The gelling behavior of SAFs could be synergistically regulated by CNC and Ca^2+^.
• CNC could be combined with SAFs by hydrogen bonding and van der Waals forces.
• CNC and Ca^2+^ changed the protein conformation and intermolecular force of SAFs gel.
• This study provided an idea to develop multi-functional SAFs gel-based products.

## 1 Introduction

Soybean protein amyloid fibrils (SAFs) is a linear protein aggregate rich in cross β structure, which is formed by self-assembly of soybean protein as raw material under conditions below isoelectric point and above denaturation temperature for a long time and driven by hydrogen bonding, van der Waals force, static electricity and other forces (Wang et al., 2020). Compared with natural soy protein, SAFs have better tolerance to extreme environments such as acid, heat and enzymes, and also act as gelling agents, emulsifiers, foaming agents, antioxidant and antibacterial active substances (Alavi, Emam-Djomeh, Mohammadian, Salami, & Moosavi-Movahedi, 2020), which makes them very suitable for the preparation of novel hydrogels for functional protein-based materials or food ingredients. At present, the development of new protein hydrogel using foodborne protein amyloid fibril as a gelation forming component is an important breakthrough in the application research and value-added transformation of protein functional structure. However, due to the different morphologies of SAFs, the mechanical properties of the gel building blocks (gel networks) themselves are low and the cross-linking is insufficient, resulting in the common problem of insufficient mechanical properties of the prepared SAFs-based hydrogels (Gu et al., 2018). Therefore, it is particularly important to achieve targeted regulation of SAFs gel properties and improve its mechanical properties.

Polyphenol modification is the main method to regulate the properties of foodborne protein amyloid fibril gels. Xu et al. found that epigallocatechin gallate (EGCG) could raise the gel properties of soybean protein amyloid fibril and the energy storage modulus of the compound gelling under a specific addition ratio is nearly eight times higher than that of the fibrillar gel (Xu et al., 2022). The above method seems to be able to achieve different degrees of improvement in gel properties by changing the protein structure of foodborne protein amyloid fibril. However, polyphenol modification has some problems such as high raw material cost, poor component stability and complex extraction process. Therefore, there is an urgent need to develop green, economical, and efficient protein modification technologies aimed at enhancing the gel performance of foodborne protein amyloid fibrils. Polysaccharide-protein interaction and salt ion treatment technology are more suitable and have great development potential.

Cellulose nanocrystals (CNC) is a cellulose-based nano-polysaccharide material, which has the characteristics of rich hydroxyl group, large specific surface area, high crystallinity and mechanical strength, non-toxicity, and good biodegradability (Mo, Huang, Yue, Hu, & Hu, 2024). A lot of studies have discovered that CNC can enhance the performance of protein gel by facilitating the cross-linking aggregation of protein molecules and acting as an active filler to absorb water and expand and fill the gaps in the gel network (Jin, Qu, Wang, Li, & Wang, 2022; Zhang et al., 2024). However, polysaccharide addition alone is not good for improving the overall gel quality, and crosslinking agents are often added during polysaccharide gelation to facilitate the formation of 3D networks. Calcium chloride (CaCl_2_) is a great crosslinker and calcium fortifying agent usually used in the food sector, which plays a crucial effect in the formation of gels: Stabilizes the gel matrix and promotes gel formation in a variety of environments, which might be attributed to electrostatic shielding of Ca^2+^ and the formation of “salt Bridges” (Guo et al., 2023). Our previous study found that CNC and Ca^2+^ can synergistically add the gel strength and viscoelasticity of pea protein isolate (Zhang et al., 2024). However, there are few reports on the regulatory effects of CNC and Ca^2+^ and their mechanisms on SAFs gel net structure and the improvement of gel properties. Moreover, the relationship between intermolecular interactions and gel properties remains unclear. It is different from the recent performance shortcomings of biodegradable biodegradable polylactic acid by introducing cellulose-based nanomaterials (Dong et al., 2025; Wu et al., 2025; Xie et al., 2025; Zhan, Abdalkarim, Yu, & Mehanny, 2025), our work aims to reveal how CNC and Ca^2+^ synergistically balance hydrogen bonding and ionic/hydrophobic interactions to construct filamentous β-sheet gel networks, thereby decoupling gel strength and water retention in amyloid fibril systems.

Therefore, this study proposes the hypothesis that the cooperative influence of CNC and Ca^2+^ can effectively regulate the gel properties of SAFs. In this study, 1) CNC and SAFs were prepared by using pear peel pomace and soybean protein as raw materials. Pear peel, as a by-product of agricultural products processing, is rich in resources, low cost, and rich in nutrients such as water, pectin and dietary fiber. Compared with bagasse, pear peel cellulose had a larger aspect ratio and was more effective in enhancing the cross-linking of the gel network when used as an “active filler”(Wu et al., 2025). However, pear peel residue is often not effectively used as a by-product of processing. The preparation of CNC from pear peel residue is based on the principles of sustainable development, waste reduction and innovation. 2) Exploring the influence of adding CNC or/and Ca^2+^ on the performance of SAFs gel. 3) Further, the regulatory mechanism of the cooperative influence of CNC and Ca^2+^ on the performance of SAFs gel was systematically elucidated from four perspectives: aggregation behavior, multi-scale structure, functional group distribution and intermolecular interaction. This study can complete the diversified control of the properties of SAFs based gels, and provide the base for the exploiting of related products.

## 2 Materials and methods

### 2.1 Materials and reagents

Soy protein isolate (SPI, purity>80 %) powder and anhydrous calcium chloride (CaCl_2_) were purchased from Shanghai Yuanye Biotechnology Co., Ltd. The Dangshan pears used in this study were purchased from local orchards in Dangshan County, Anhui Province. To ensure freshness, the fruits were immediately stored at 4 °C after harvesting, with a storage duration not exceeding 72 h. All pears selected for the experiment had a diameter of 7–8 cm, a unit weight of 250–300 g, and were free from diseases, pests, and mechanical damage to guarantee sample uniformity. All other chemicals used, such as sodium hypochlorite and 2-mercaptoethanol, were of analytical grade and were procured from Macklin Biochemical Technology Co., Ltd. in Shanghai.

### 2.2 Preparation and characterization of CNC

The CNC used in this study was made from pear peel residue with reference to acid hydrolysis (Xiao et al., 2019). The PPC was obtained from the pear peel pomace (PPP) after fat removal, alkali treatment and bleaching treatment. The PPC was mixed with sulfuric acid solution (60 %, *w*/w), centrifuge the acid-hydrolyzed CNC suspension at 8000 g and 4 °C for 10 min. Discard the supernatant, resuspend with deionized water, and repeat centrifugation and washing 5 times until the pH of the supernatant is neutral. This would yield the purified CNC suspension. Atomic force microscope (AFM, Brock AG, Karlsruhe, Germany) was used to detect the morphological features of CNC, and Nano Measurer 1.2 software was used to analyze the size of AFM images. The potential value of the CNC was recorded using the Nano particle potential analyzer (ZS90, Malvern, UK). Fourier infrared spectroscopy was used to document the FTIR spectra of the above samples in the area of 400–4000 cm^−1^, and the functional group changes of the samples were analyzed. The thermal stability of the sample was analyzed by heating it from 30 °C to 600 °C at a heating ratio of 10 °C/min using the thermogravimetric analyzer (TGA 55, TA Instruments, USA). X-ray diffractometer (XRD, SmartLab SE, Rigaku, Japan) was used to scan the sample at an area of 5 to 90° (2θ) and a speed of 10°/min.

### 2.3 Preparation and characterization of SAFs

The preparation of SAFs was based on the original method and was modified accordingly (Zhou et al., 2024). SPI was completely dissolved in water using a solid-liquid ratio of 1:10, followed by adjustment of the pH to 2.0 with 6 mol/L hydrochloric acid. Subsequently, magnetic stirring was applied for 30 min at room temperature. The liquid obtained by centrifugation (10,000 *g*, 4 °C, 30 min) was collected and stirred in a water bath at 85 °C for 20 h. After cooling to room temperature, the resultant SAFs suspension was stored at 4 °C for future use, the sealed storage time should not exceed 7 days.

The microscopic morphology of SAFs was observed by atomic force microscopy, and the size analysis of AFM images was performed by Nano Measurer 1.2 software. Fluorescence spectrophotometer (FL 6500, PerkinElmer, USA) was used to detect the Thioflavin T (Th T) fluorescence intensity of the sample with excitation wavelength of 440 nm and emission wavelength of 490 nm. X-ray diffractometer (XRD, SmartLab SE, Rigaku, Japan) was used to scan the above freeze-dried samples in Cu-Kα radiation mode (λ = 0.154 nm) at a scanning range of 5–90° (2θ) and a scanning speed of 10°/min. Using the Fourier infrared spectrometer (Vertex 70, Bruker, Germany) record freeze-dried samples of protein (SPI and SAFs) in the range of 400–4000 cm^−1^ FTIR spectrum. The surface hydrophobicity of 0.005 mg/mL SPI and SAFs was detected by fluorescence spectrophotometer (FL 6500, PerkinElmer, USA) using 8-aniline-1-naphthalene sulfonic acid as a fluorescence probe with an excitation wavelength of 380 nm, an emission wavelength range of 400–700 nm, and an emission slit of 10 nm.

### 2.4 Preparation of SAFs-based composite gels

The preparation of pea protein composite gel was based on the original method and was appropriately revised on this basis (Zhang et al., 2024). According to the consequence of the early optimization test, 0.09 % (*w*/*v*) CNC (meaning 0.09 g of CNC per 100 mL of solution) and 400 mmol CaCl_2_ (meaning 0.04 mol of CaCl₂ per liter of solution) were added to the SAFs solution alone or in combination (the ultimate protein concentration was 10 mg/mL). The resulting sol-solution was stirred for 12 h at room temperature, then divided into 10 mL beakers and sealed. The small beaker was heated in a water bath, gradually increasing the temperature from 25 °C to 95 °C, and then maintained at a constant temperature for 30 min. Following this, it was immediately cooled down to room temperature and subsequently stored at 4 °C for 12 h to obtain the SAFs composite gel. The four groups of gel samples were named SAFs (SAFs-based gels without CNC and CaCl_2_), SAFs/CNC (SAFs-based gels with only CNC), SAFs/CaCl_2_ (SAFs-based gels with only CaCl_2_), and SAFs/CNC/CaCl_2_ (SAFs-based gels with both CNC and CaCl_2_).

### 2.5 Gel performance test

#### 2.5.1 Rheological behavior

The samples were detected using a hybrid rheometer (Discovery HR-1, TA Instruments, USA). A parallel steel plate with a diameter of 40 mm was employed for the detection process and the rheological curves of the samples at the 1000 μm loading gap were recorded by frequency scanning and flow scanning, respectively. Frequency sweep: test temperature 25 °C, angular frequency range 0.1 to 100 rad/s, Strain set at 1 %. Flow scanning: Performed at 25 °C with shear rates ranging from 0.1 to 100 rad /s.

#### 2.5.2 Texture profile analysis

Gel samples were tested using a texture analyzer (TA.XT.Plus, Stable Micro Systems Co., UK). The pre-test speed was 2.0 mm /s, the test speed was 1.0 mm /s, the test speed was 2.0 mm /s, the test distance was 5.0 mm, the recovery height was 40 mm, the recovery speed was 10 mm /s, the contact recovery force was 0.2 g. The gel strength is represented by the slope worth (K) of the linear correlation between the forces of different specimens and their deformation distances.

#### 2.5.3 Water holding capacity (WHC)

The gel sample was centrifuged at 10000 *g*, 4 °C, 15 min, and then drained upside down for 5 min. Record M_0_, M_1_ and M_2_, and calculate WHC using eq. (1).

(1) $$\mathrm{WHC}=\frac{M_{2}-M_{0}}{M_{1}-M_{0}}\times 100\%$$

M_0_: the mass of empty centrifuge tubes (g).

M_1_: the total mass of the empty centrifuge tube and the gel sample before centrifugation (g).

M_2_: after centrifugation and inversion for 5 min to drain off the liquid, the total mass of the empty centrifuge tube and the gel sample (g).

#### 2.5.4 Water distribution of gel

Samples were measured by Low-field NMR and MRI testing analyzer (MesoMR23-060H–I, Niumag Electric Co., China), and the hydrogen proton density map was acquired using the imaging software Numag NMR Imaging System V 3.0, and the images were processed Image processing by pseudo color software.

### 2.6 Protein molecular morphology test

#### 2.6.1 Turbidity

The sample was diluted 500-fold and shaken thoroughly. The absorbance value at 600 nm (OD600) was documented by a microplate reader (VICTOR Nivo, PerkinElmer, USA).

#### 2.6.2 Dissolubility

The samples were diluted 600 times with deionized water and then centrifuged at 10000 *g* for 30 min at 4 °C to collect the supernatant. The protein concentration of supernatant at 595 nm was determined by using the protein concentration assay kit and the microplate reader (VICTOR Nivo, PerkinElmer, USA). Solubility was expressed as the percentage (%) of the protein concentration in the supernatant relative to the total protein concentration in the colloidal sample.

#### 2.6.3 Protein subunit distribution

After diluting the protein solution to 1 mg/mL, it was mixed with the sample buffer in a 1:1 ratio and heated in a water bath at 100 °C for 2 min. Subsequently, electrophoresis was performed at 80 V using an electrophoresis apparatus (DYCZ-24DN, Beijing Liuyi Biotechnology Co., LTD., China) until the end. During the experiment, Coomassie bright blue dye solution was used for dyeing, the duration was 1 h, and the decolorization process was carried out with decolorization solution every 1 h until the electrophoretic bands were clearly visible. Finally, electrophoretic images were collected by the gel imaging system (Bio-Rad Universal Hood II, USA), and the obtained electrophoretic images were analyzed and processed by Image Lab 3.0 image processing software.

### 2.7 Gel structure test

#### 2.7.1 Protein secondary structure

The ground samples were pressed into thin sheets with spectrally pure KBr powder using a tablet press (sample:KBr = 1:100). Absorbance was recorded by an infrared spectrometer with a real spectral area of 400–4000 cm^−1^, scanned 32 times in transmission mode. The spectral results in the amide I band (1600–1700 cm^−1^) were processed using PeakFit v4.12 software to analyze the protein secondary structure.

#### 2.7.2 Crystal structure

The experimental parameters were 5–90° (2θ), 5°/min, 40 kV, 40 mA, and X-ray diffractometer was used to obtain the XRD patterns of the samples.

#### 2.7.3 Conformational stability

Set parameters: 10 °C/min, 30–600 °C, the thermal stability of the sample was assessed using a thermogravimetric analyzer (TGA 55, TA Instruments, USA).

#### 2.7.4 Gel network structure

After freeze-drying and vacuum gold-plating for 60 s, the sample's microstructural details were captured using a scanning electron microscope (SEM, S-4800, Hitachi, Japan).

### 2.8 Functional group test

#### 2.8.1 Total sulfhydryl

The gel sample was weighed, added to normal saline and magnetically stirred under ice bath conditions. After centrifuging the sample at a speed of 2500 rpm for 10 min, the supernatant was obtained for further examination. The sample's absorbance at 405 nm was determined by a micro-total sulfhydryl test box (Nanjing established the Institute of Bioengineering, Nanjing, China).

#### 2.8.2 Surface hydrophobicity

The fluorescent probe was obtained by dissolving 2.4 mg of 8-aniline-1-naphthalenesulfonic acid in 200 μL DMSO and adding water to 2 mL. The fluorescent probe was mixed thoroughly with the solution sample and reacted in the dark for 10 min. The surface hydrophobicity of the sample was detected using a fluorescence spectrophotometer (FL 6500, PerkinElmer, USA) with the following settings: an excitation wavelength of 380 nm and an emission wavelength ranging from 400 to 700 nm.

#### 2.8.3 Amino acid residue microenvironment

*(1) Ultraviolet spectrum*

The 0.1 mg/mL protein solution was detected by ultraviolet spectrophotometer (UV-2600i, Shimadzu, Japan) with a wavelength of 220–400 nm and a data interval of 0.2 nm, and the second derivative map was processed by Origin Pro 2023 software.

*(2) Synchronous fluorescence*

Fluorescence spectrophotometer (FL 6500, PerkinElmer, USA) was used to detect the changes in the amino acid microenvironment of tryptophan (Δλ = 15 nm) and tyrosine (Δλ = 60 nm) in 0.05 mg/mL protein solution. The excitation wavelength ranged from 200 to 400 nm, the wavelength interval Δλ was 15 nm or 60 nm, the excitation slit was 5 nm, and the emission slit was 5 nm.

*(3) Three-dimensional fluorescence*

The three-dimensional fluorescence intensity of 0.05 mg/mL protein solution was scanned with a fluorescence photometer (FL 6500, PerkinElmer, USA). Experimental parameters: excitation wavelength 190 nm, scanning 32 times, excitation wavelength interval 5 nm. The initial emission wavelength was 200 nm, the final emission wavelength was 500 nm, and the emission slit was 5 nm. Both the excitation slit and emission slit were 5 nm.

### 2.9 Molecular interaction tests

#### 2.9.1 Intermolecular forces

Five kinds of solutions that can destroy the intermolecular force were prepared, and 1 g gel sample was fully mixed with the above 5 mL solution. After standing at 4 °C for 1 h, the liquid after centrifugation was collected (4 °C, 10000 g, 15 min) and the protein concentration was determined. Detailed references to experimental methods (Xue et al., 2017).

#### 2.9.2 Molecular docking

For conducting molecular docking studies with AutoDock software, ligands in the form of CNC molecules were sourced from the PubChem database (https://pubchem.ncbi.nlm.nih.gov), whereas the molecular structure of SAFs (PDB ID:2BFI), serving as the docking receptors, was retrieved from the protein database (http://www.rcsb.org). During the docking process, the optimal conformation was visually analyzed using Pymol 2.2.0 software and Discovery Studio 4.5 software.

### 2.10 Data statistics and analysis

The experiments were conducted in triplicate or greater (*n* ≥ 3), and the results are reported as the mean ± standard deviation. Univariate Analysis of variance (ANOVA) and Tukey test were used to analyze the significance of experimental data using statisticx 8.0 software, the significance level is set at α = 0.05. In order to explore the correlation among indicators, Pearson correlation analysis method was used and Origin Pro 2023 software was used to draw relevant analysis charts.

## 3 Results and analysis

### 3.1 Physical and chemical characteristics of CNC

**Fig. 1:**
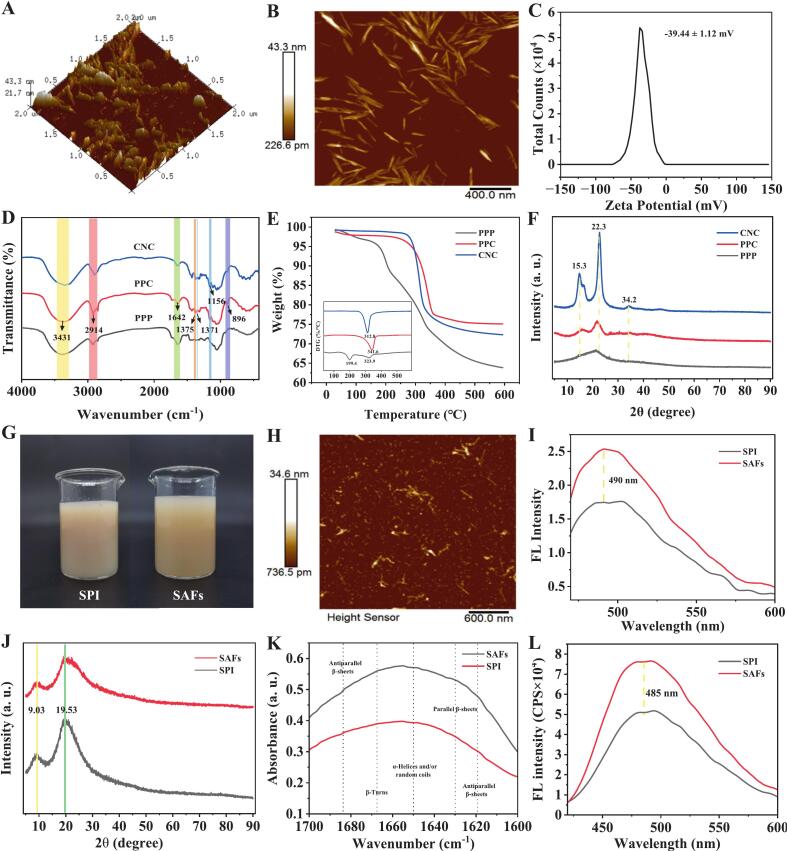
(A-B) Atomic force microscopy image, (C) Zeta potential, (D) FTIR spectroscopy, (E) TGA and DTG curves, and (F) X-ray diffraction pattern of CNC originated from pear peel pomace. PPP: pear peel pomace, PPC: pear peel pomace cellulose, CNC: cellulose nanocrystal. (G) Macroscopic morphology, (H) atomic force microscopy image, (I) ThT fluorescence diagram, (J) X-ray diffraction pattern, (K) FTIR spectroscopy, and (L) surface hydrophobicity of SAFs. SPI: soy protein isolate, SAFs: soybean protein amyloid fibrils.

From the AFM images (Fig. 1A-B), it can be seen that the pear peel residue source CNC displays a classic rod-shaped structure, and its average length, diameter, and length-diameter rate were 157.67 ± 50.94 nm, 22.04 ± 5.31 nm, and 7.96 ± 2.32, respectively (Fig. S1). The nanocellulose suspension is deemed stable when the Zeta potential value falls below −30 mV or exceeds either 30 mV or 25 mV. (Xiao et al., 2019). Accordingly, the CNC suspension of pear peel pomace prepared in this study (−39.44 mV) showed good dispersion stability (Fig. 1C).

The FTIR results (Fig. 1D) showed that the bands associated with the characteristic structure of cellulose, such as those at 1375 cm^−1^ (C—H bending), 1156 cm^−1^ (unsymmetrical stretching of C-O-C glycosidic bonds), and 896 cm^−1^ (C—H oscillating vibration of glycosidic bonds), became more intense following the extraction and separation treatments. This was attributable to the efficient elimination of non-cellulose components and the adequate exposure of fiber components. The large surface area of CNC helped it absorb more heat. In addition, the sulfate groups introduced in the acid hydrolysis process reduced the thermal degradation activation energy of CNC, resulting in a lower maximum thermal degradation temperature of CNC (312.8 °C) than that of PPC (341.6 °C) (Fig. 1E). XRD diagram is shown in Fig. 1F, diffraction peaks of the characteristic crystal face of cellulose type I were observed, including 15.3° (110 plane), 22.3° (002 plane), and 34.2° (004 plane) (Xu et al., 2023). After extraction and separation treatment, the main crystal peak at 22.3° became more intense and narrowed considerably, and the crystallinity increased from 43 % (PPC) to 84 % (CNC), indicating the effective removal of amorphous regions. In summary, CNC with rod-like structure, good dispersion stability and high crystallinity was successfully prepared from pear peel pomace.

Cellulose nanocrystals derived from pear peel residue (P-CNC) exhibit significant differences in core characteristics, such as surface charge, morphology, and crystallinity compared with those sourced from wood (W-CNC) or cotton (C-CNC). Although all types of CNC retain the cellulose I crystal structure and typical rod-like morphology, enabling them to form basic hydrogen bonds with hydroxyl or amide groups in proteins, P-CNC demonstrate superior dispersibility in solution due to their higher absolute zeta potential value (−39.44 mV), which reduced aggregation and promoted the formation of a more uniform composite gel network with proteins (Rahbar Shamskar, Heidari, & Rashidi, 2016). The aspect ratio of P-CNC (7.96 ± 2.32) falls between that of W-CNC (5–8) and C-CNC (∼7.69), and their improved dispersibility facilitates more efficient physical entanglement with proteins (Mo et al., 2024; Zhao et al., 2023). As an active filler, P-CNC not only filled the pores of the protein gel network and interact with hydrophobic domains of proteins via van der Waals forces, but their slightly lower crystallinity and potential traces of residual polyphenols or pectin could also provide additional functional groups. These groups might enhance secondary interactions, such as hydrogen bonds or π–π stacking, with aromatic amino acids in proteins (Wei et al., 2019). It is worth noting that although these surface characteristic differences might modulate the binding strength, the fundamental mechanism, where CNC interact with proteins via hydrogen bonding through hydroxyl groups remained consistent across different sources. This study successfully validated the feasibility of a “waste-to-value” strategy using pear peel residue, without altering the core interaction mechanism between CNC and proteins.

### 3.2 Physical and chemical properties of SAFs

After fibrotic treatment, the SPI solution changed from the original beige to brownish-yellow (SAFs) (Fig. 1G), which was speculated to be due to changes in protein molecular morphology. SAFs exhibited a characteristic slender rod-like morphology (Fig. 1H), possessing an average length of 155.77 ± 63.43 nm, a diameter of 20.52 ± 6.15 nm, and an aspect ratio of approximately 7.45 ± 3.51 (Fig. S2, *n* = 50). After combining with ThT, SAFs exhibited higher fluorescence intensity at the characteristic peak of 490 nm (Fig. 1I). This was due to the alteration in the conformation of the ThT molecule upon binding to the β-folded structure of SAFs, which allowed for internal charge transfer, resulting in a significant increase in fluorescence strength (Arad, Green, Jelinek, & Rapaport, 2020).

It could be seen from the XRD pattern that SAFs and SPI only had obvious crystal diffraction peaks at 9.03° and 19.53° (Fig. 1J). Among them, 9.03° corresponded to the crossed β structure vertical to the fibril axes, and the segment distance was 8.99 Å. 19.53° corresponded to the β-folded polypeptide skeleton parallel to the fibril axes, and the chain distance from the second peak was 4.57 Å. These two main peaks confirmed that the primary protein secondary structure in SAFs was the crossed beta-folded conformation (Gao et al., 2023). The results of FTIR were further validated (Fig. 1K), SAFs contained a low proportion of α-helical structures, β-turns, and random coil conformations and high proportions of parallel and antiparallel β-fold conformations (*p* < 0.05) compared to SPI (Table 1). Compared with SPI, the 8-aniline-1-naphthalene sulfonic acid fluorescence peak of SAFs at around 485 nm was significantly enhanced (Fig. 1L). This might be because the amino acid polarity microenvironment of SAFs was changed after fibrotic treatment, and the hydrophobic groups located within the protein structure were exposed to the surrounding hydrophilic environment, resulting in increased contact probability between the polar water environment and the hydrophobic groups on the surface of protein. In summary, SAFs with high surface hydrophobicity and rich cross-β-fold structures were successfully prepared.

**Table 1:** Protein secondary structure percentage of SPI and SAFs.

Secondary structure	Wavelength range(cm^−1^)	Relative contribution (%)	
		SPI	SAFs
β-Turns	1668	23.66 ± 0.28^a^	21.64 ± 0.54^b^
α-Helices and/or random coils	1650	26.89 ± 0.95^a^	24.32 ± 0.87^b^
Parallel β-sheets	1630	21.00 ± 0.45^b^	22.80 ± 0.52^a^
Antiparallel β-sheets	1619/1683	28.45 ± 1.06^b^	31.24 ± 1.13^a^

Different letters (a-b) indicate statistically significant differences between SPI and SAFs (*p* < 0.05).

### 3.3 Gel properties

#### 3.3.1 Rheological behavior

**Fig. 2:**
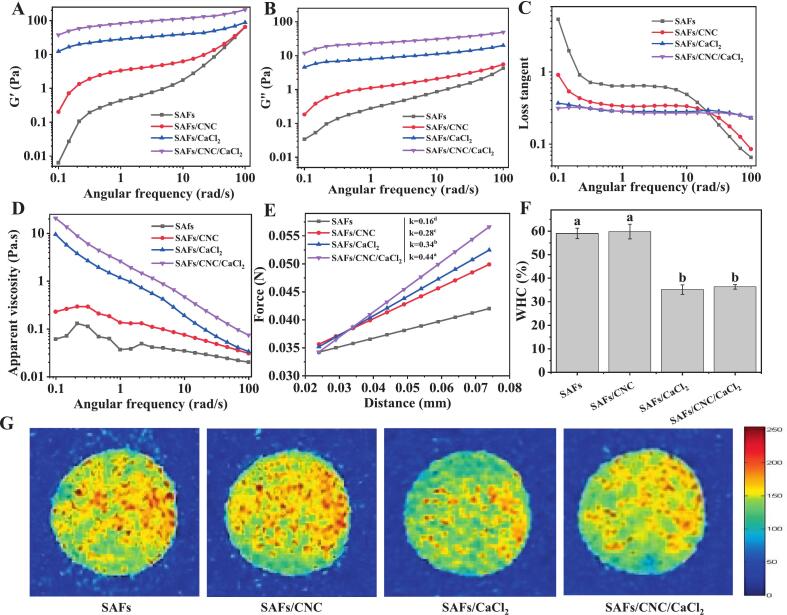
(A) Storage modulus G′, (B) loss modulus G′′, (C) loss tangent curve, and (D) apparent viscosity of SAFs-based composite sols. (E) Texture profile analysis curve, (F) WHC, and (G) hydrogen proton density plot of SAFs-based composite gels. Different letters (a-d) indicate significant differences between the different treatment groups (*p* < 0.05).

With increasing angular frequency, the G′ and G′′ values of the SAFs-based gel gradually increased (Fig. 2A-B). At the same angular frequency, the elasticity and viscosity of SAFs composite gel were bigger than that of the control group, and the viscoelasticity of the CNC and CaCl_2_ cooperative treatment group was the largest. CNC was characterized by its large specific surface area and small size, which could effectively raise the structural stability of the gel network as an active filler. (Zhang et al., 2024). Ca^2+^ may have more enhanced the degree of cross-linking between gel components by establishing “salt bridges”. At the same angular frequency, the G′ value of the SAFs composite gel exceeded that of G′′. Moreover, in the same angular frequency range, the loss Angle tangent value (Tan δ) of the composite gel was less than 1 (Fig. 2C), indicating that the CNC and/or CaCl_2_ strengthened SAFs gels exhibit solid-like elastic behavior, which may be related to the formation of 3D gel structures with high mechanical strength.

As the shear rate increased, the viscosity of the gel demonstrated a declining trend (Fig. 2D), showing the shear-thinning properties of non-Newtonian fluid. Under equal shear ratio, the apparent viscosity of SAFs based composite gel was higher than that of SAFs gel, and the viscosity of the composite gel added with CNC and CaCl_2_ was the largest. Zhao et al. noticed that the viscosity of myofibrillar protein composite gel increased remarkably after the addition of regenerated cellulose (*p* < 0.05), because the regenerated cellulose as a filler could fill the gap in the gel structure and make the gel denser. In addition, the regenerated cellulose itself could form a network gel in the protein matrix, which made the myofibril composite gel have higher viscosity (Zhao, Zhou, & Zhang, 2019). However, metal ions (K^+^, Ca^2+^, etc.) formed larger aggregates by promoting aggregation of egg yolk proteins, which led to restriction of movement of protein molecules (Li et al., 2024).

#### 3.3.2 Gel strength

TPA in Fig. 2E showed that the mechanical constants of SAFs-based gels enlarged from 0.16 × 10^−3^ N/m (SAFs) to 0.28 × 10^−3^ N/m (SAFs/CNC), 0.34 × 10^−3^ N/m (SAFs/CaCl_2_) and 0.44 × 10^−3^ N/m (SAFs/CNC/CaCl_2_), respectively. This proved that the single and combined adding of CNC and CaCl_2_ could efficiently enhance the gel strength of SAFs, and the combined treatment of both showed a synergistic strengthening effect. Some studies had shown that CNC could facilitate the cross-linking of SAFs protein, causing the gel component unit, the 3D gel network, to become stronger (Zhou et al., 2024). The addition of Ca^2+^ created “salt bridges” and electrostatic shielding function, which accelerated the aggregation behavior of protein molecules and increased probability of interactions between protein molecules, thus more improving the gel intensity (Yan et al., 2022).

#### 3.3.3 Water holding capacity (WHC)

Compared with SAFs gels, the WHC value of SAFs/CNC gels was added by 1.32 % (*p* > 0.05), and the weak increase may be related to the hydrogen bond interaction between the many hydroxyl groups on the surface of CNC and water molecules. However, the WHC value of SAFs/CaCl_2_ and SAFs/CNC/CaCl_2_ gels decreased by 40.39 % and 38.47 %, respectively (Fig. 2F), showing that Ca^2+^ impaired the adsorption and retention ability of SAFs gels for free water molecules. The addition of Ca^2+^ first induced the rapid aggregation of protein molecules. This process restricted the interaction between proteins and water molecules, which in turn triggered the formation of a gel network with uneven pores and a relatively rough structure. This network structure directly weakened the “trapping” and “retaining” capabilities of the composite gel for free water(Zhang et al., 2024). Meanwhile, Ca^2+^ also formed “salt bridges” with the carboxyl groups on the surface of SAFs. This action competed for the hydrogen bond binding sites between SAFs and water molecules, ultimately destroying the original hydrogen bond network of SAFs. In addition, Ca^2+^ promoted the formation of thick sheet-like aggregated structures in SAFs. Although such structures enhanced the overall strength of the gel, they compressed the “bound water space” within the gel network, causing part of the originally bound water to be converted into free water.

#### 3.3.4 Water distribution

According to the NMR images in Fig. 2G, the hydrogen proton density in the detection region of SAFs/CNC gel was the highest, while that of SAFs/CaCl_2_ gel was the lowest. It could be seen that CNC raised the percentage of bound water or stagnant water in SAFs gel and reduced the percentage of free water, that is, CNC stabilized the water phase in SAFs gel and limited the free movement of water, while Ca^2+^ showed the opposite effect. It has been reported that sodium carboxymethyl cellulose could increase the pore density of the soybean protein isolate gel network, thereby “trapping” more free water molecules (Sun et al., 2024). Therefore, we speculated that the enhanced red response signal of SAFs/CNC gels might have been due to the formation of complex gel network structural units with a strong capacity to maintain water molecules, induced by CNC. However, the presence of Ca^2+^ ions hindered the attachment of hydrophilic protein groups to water molecules, thereby diminishing the overall hydrophilic nature of the gel system and leading to an increase in free water content within the gel (Xiao et al., 2021).

In summary, the synergistic modulation strategy proposed in this study enabled the customizable design of SAFs gel properties. The results demonstrated that CNC and CaCl₂ acted synergistically to enhance the viscoelasticity and gel strength of SAFs-based gels, while CaCl₂ alone reduced their water holding capacity. For food products requiring good chewiness and structural stability, such as plant-based sausages and restructured meat products, high gel strength improved elasticity and sliceability, whereas appropriately modulated water holding capacity helped prevent texture deterioration caused by moisture exudation during processing or storage. Therefore, by flexibly adjusting the addition ratios of CNC and Ca^2+^, a range of gel products ranging from “soft, low-water-holding” to “firm, high-strength types” could be achieved. This approach provided a new strategy for the targeted design of multifunctional SAFs gels and offered novel insights into quality control for SAFs-based gel products.

### 3.4 Protein aggregation behavior

#### 3.4.1 Turbidity and solubility

**Fig. 3:**
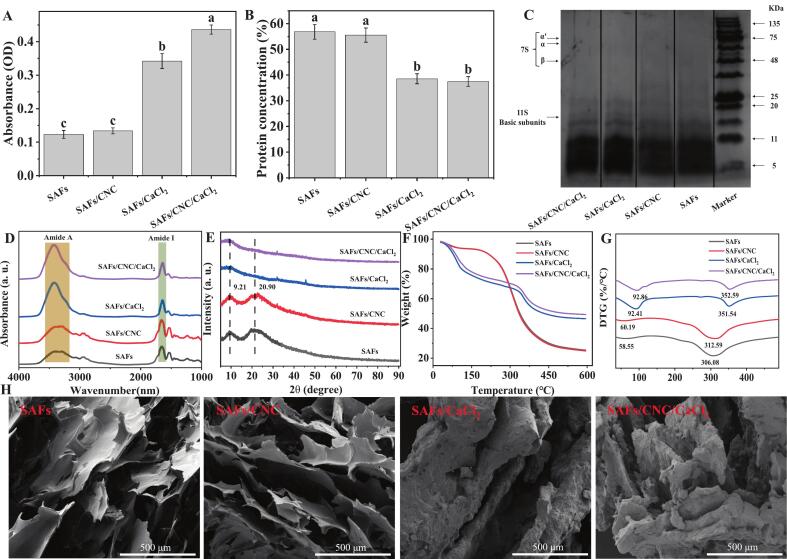
(A) Turbidity, (B) Solubility, (C) SDS-PAGE images, (D) FTIR spectroscopy, (E) X-ray diffraction pattern, (F) TGA curves, (G) DTG curves, and (H) microstructure plots of SAFs-based composite samples. Different letters (a-c) indicate significant differences between the different treatment groups (*p* < 0.05).

As could be seen from Fig. 3A, compared with SAFs solution, the turbidity of SAFs/CNC solution had no remarkable changing (*p* > 0.05), while the turbidity of SAFs/CaCl_2_ and SAFs/CNC/CaCl_2_ solutions raised by 177.34 % and 253.49 %, respectively (*p* < 0.05). The findings indicated that Ca^2+^ promoted the formation of large aggregates in SAFs solution. The addition of Ca^2+^ produced “salt bridges”, and cross-linking between the “salt bridges” facilitated the formation of more protein aggregates, leading to increased turbidity (Deng et al., 2020). The turbidity increased further after adding CNC to the SAFs/CaCl_2_ solution (*p* < 0.05). This might have been due to the charge neutralization of a lot of negatively charged sulfate groups carried on the surface of CNC with calcium ions or positively charged amino acid residues, which changed the electrostatic force of protein molecules, resulting in flocculation. The presence of calcium ions also changed the solubility of SAFs (Fig. 3B).

Compared with SAFs, the solubility of SAFs/CNC was reduced by 2.22 % (*p* > 0.05), while the solubility of SAFs/CaCl_2_ and SAFs/CNC/CaCl_2_ were decreased by 32.13 % and 34.02 % (*p* < 0.05), respectively. This might have been due to the salting-out role caused by the addition of high concentrations of Ca^2+^ (Wang, Yang, Li-Sha, & Chen, 2021), which competed with protein molecules in solution for free water, thereby disrupting the hydration of proteins and causing a decrease in protein solubility.

#### 3.4.2 Protein subunit distribution

As shown in Fig. 3C, SAFs only formed small molecular weight protein subunit bands in the range below 20 kDa. This was consistent with Gao et al.'s finding that no clear bands were observed in the 20–135 kDa range of SAFs, indicating that the 7S-α’, 7S-α, 7S-β, and 11S-basic subunits in SPI underwent acid hydrolysis during fibrosis to form protein subunits with smaller molecular weights (Gao et al., 2023). The adding of CNC resulted in a lighter color of the 11 kDa electrophoresis strip in SAFs, and the optical density value of SAFs/CNC at this strip decreased by 9.06 %. The addition of Ca^2+^ deepened the color of the band, and the optical density of SAFs/CaCl_2_ and SAFs/CNC/CaCl_2_ raised by 27.14 % and 17.71 %, respectively. On one hand, this might be because the abundant hydroxyl groups and rigid rod-like structures in the CNC could physically prevent some aggregated subunits from further combining, introducing spatial resistance. On the other hand, calcium ions neutralized the negative charges of the protein side chains, reducing electrostatic repulsion, and accelerating cross-linking. When both were present simultaneously, the spatial obstruction and electrostatic shielding work together, causing the aggregation of certain protein subunits to become more compact, while also limiting the stability of smaller subunit bands (such as protein components with a molecular weight of approximately 11 kDa) (Zhao, Xu, Yuan, Qi, & Li, 2024).

### 3.5 Multi-scale structure

#### 3.5.1 Protein secondary structure

The amide A region, characterized by broad peaks ranging from 3250 cm^−1^ to 3550 cm^−1^, corresponds to O—H tensile vibrations present in proteins or water molecules. Compared with SAFs, the peak acreage and strength of the amide A region of the composite gel after the adding of CNC or/and CaCl_2_ were added to varying degrees (Fig. 3D), suggesting that CNC or/and CaCl_2_ may enhance the intermolecular hydrogen bond interaction.

Compared with SAFs gels, the adding of CNC marginally added the ratio of β-fold, β-turn, and intermolecular β-fold of gel proteins, and significantly decreased the relative level of α-helix (Table 2). Compared with SAFs, the contents of β-sheet in SAFs/CaCl_2_ and SAFs/CNC/CaCl_2_ gels increased by 34.45 % and 39.97 %, respectively, while the contents of random coiling, α-helix, β-corner and intermolecular β-fold decreased by 22.48 % and 22.94 %, 22.60 % and 24.15 %, 12.33 % and 17.31 %, 13.76 % and 9.39 %, respectively. This indicated that the addition of CNC and/or Ca^2+^ caused the α-helix in the SAFs gel to unfold and convert to β-folding, resulting in varying degrees of growth in the proportion of β-folding. The above findings were similar to found by Pan et al., who reported that the addition of Ca^2+^ induced a decline in the proportion of α-helices and a growth in the proportion of β-folds in the secondary structure of K-carrageenan myosin gels (Pan, Guo, Li, Song, & Ren, 2017). Research has indicated that a decrease in α-helix structure and an increase in β-folding enhance the ability of proteins to form thermal gels, thereby improving the gel's properties (Zhao et al., 2024). Therefore, it was concluded that the growth in β-fold relative content in SAFs composite gels may have been a significant factor in making the composite gels stronger and more stable.

**Table 2:** Protein secondary structure percentage of SAFs-based composite gels.

Protein secondary structure	β-sheet (%)	Random coil (%)	α-helix (%)	β-turn (%)	Intermolecular β-sheet (%)
Wavelength sample	1600–1640 cm^−1^	1640–1650 cm^−1^	1650–1660 cm^−1^	1660–1700 cm^−1^	1620–1630 cm^−1^
SAFs	34.02 ± 0.25^c^	17.61 ± 0.09^a^	17.39 ± 0.02^a^	30.97 ± 0.18^a^	13.74 ± 0.09^a^
SAFs/CNC	34.34 ± 0.06^c^	17.39 ± 0.01^a^	17.27 ± 0.01^b^	30.99 ± 0.06^a^	13.88 ± 0.01^a^
SAFs/CaCl_2_	45.74 ± 0.26^b^	13.65 ± 0.04^b^	13.46 ± 0.03^c^	27.15 ± 0.28^b^	11.85 ± 0.07^c^
SAFs/CNC/CaCl_2_	47.62 ± 0.24^a^	13.57 ± 0.04^b^	13.19 ± 0.03^d^	25.61 ± 0.24^c^	12.45 ± 0.08^b^

Different letters (a-d) indicate statistically significant differences in the same column (*p* < 0.05).

#### 3.5.2 Crystal structure

SAFs and SAFs/CNC had obvious characteristic peaks at 9.21° and 20.90° (Fig. 3E). The position and intensity of SAFs characteristic peaks did not change meaningfully after the adding of CNC. It may have been that CNC had no effect on the formation of SAFs crystal zone. But the adding of Ca^2+^ significantly declining the peak strength of the diffraction peak at 9.21° and almost disappeared the characteristic peak at 20.90°. This may be attributed to the Ca^2+^ induced reconfiguration of the molecular structure of SAFs, leading to the formation of a new amorphous conformation characterized by low crystallinity. Similar findings were found in the research of Cao et al., in which the characteristic peaks of SAFs disappeared and crystallinity decreased after the addition of divalent cations (Zn^2+^ or Mg^2+^) (Cao et al., 2024). Ca^2+^ induced the aggregation and binding of SAFs to form intensive compound that covered the natural crystalline region of the protein, or that Ca^2+^ promoted the transformation of the protein from a crystalline structure to an amorphous region, thereby reducing the crystallinity of the SAFs gel.

#### 3.5.3 Conformational stability

The thermogravimetric (TG) curve and thermogravimetric differential (DTG) curve can reflect the conformational thermostability (Fig. 3F-G). The degradation process could be roughly classified into two period, with the first period involving the evaporation of gel-bound water and occurring below 150 °C. At this stage, the maximum thermal degradation temperature rose from 58.55 °C for SAFs to 60.19 °C for SAFs/CNC, 92.41 °C for SAFs/CaCl_2_, and reached 92.86 °C for SAFs/CNC/CaCl_2_, indicating that the adding of CNC or/and Ca^2+^ may have improved the retention capacity of SAFs-based gels for bound water. Weightlessness mainly occurred in the second stage (150–600 °C), involving thermal decomposition and evaporation of proteins and CNC. After adding the CNC (or CaCl_2_), the thermal stability of the composite gel was significantly raised, and the peak thermal degradation temperature of the gel was 312.59 °C (or 351.54 °C), respectively. Especially after the simultaneous addition of CNC and CaCl_2_, the maximum thermal degradation temperature was further increased to 352.59 °C, showing a synergistic improvement on the thermal stability of the gel. Some studies had confirmed that there might have been a particular level of combination between CNC and whey protein isolate, which could facilitate the formation of hydrogen bonds in the gel network structure, strengthen the cross-linking between CNC and protein, and enhance the thermal stability of the composite gel (Xiao et al., 2020). Ca^2+^ may have improved the conformation of SAFs by forming “salt bridges” and accelerated the aggregation of protein, thus promoting the formation of a stable network system of gel and enhancing the thermal stability (Wang et al., 2018).

#### 3.5.4 Gels network structure

There were loose and irregular lamellar structures in the SAFs gel, and the gaps (distances between lamellar structures) of the lamellar structures were not uniform, suggesting that the protein molecules were not adequately cross-linked (Fig. 3H). Studies have shown that the gap is a water channel generated by water seepage during the thermal denaturation of proteins, and the form and number of water channels can affect the formation of networks and gel WHC. The addition of CNC not only enhanced the overall three-dimensional sense of the SAFs gel, but also made the gel more compact, and there were more connections and crosslinks between the gaps of the sheet structure, and the SAFs molecules were fully unfolded. It might have been that the CNC acted as stuffings to fill the structure of the protein network, dividing the gel into “small rooms” to trap water. At the same time, CNC with a large specific surface area was also able to bind water molecules and absorb water in proteins, resulting in a larger relative concentration of the SAFs solution (“concentrated” protein matrix), thereby increasing gel intensity and WHC (Zhuang et al., 2017).

Compared with SAFs, the gel stereoscopic network structure of SAFs/CaCl_2_ and SAFs/CNC/CaCl_2_ was significantly changed, the lamellar structure was thickened, and the aggregation of protein molecules was observed, which implies a stronger protein-protein interaction. However, the gaps became significantly larger, the surface became dense, and the water in the gel system could flow more easily through these gaps, weakening the water storage capacity. This may have been due to the fact that Ca^2+^ could produce an electrostatic shielding effect, and the charge of the protein was shielded. This results in an increased probability of protein aggregation and an enlargement of aggregate size. These irregular aggregations affected the stereoscopic network structure of the gel (Wu, Hua, Chen, Kong, & Zhang, 2017). This might have also explained why the gel's performance was enhanced after adding Ca^2+^, while the WHC was poor. When both CNC and Ca^2+^ were added to the gel structure alone, significant protein aggregation but smaller gaps were also observed, reducing water loss to a certain extent. This might have been due to the fact that CNC had the characteristics of improving the WHC of the gel.

### 3.6 Functional groups of protein

#### 3.6.1 Total sulfhydryl

**Fig. 4:**
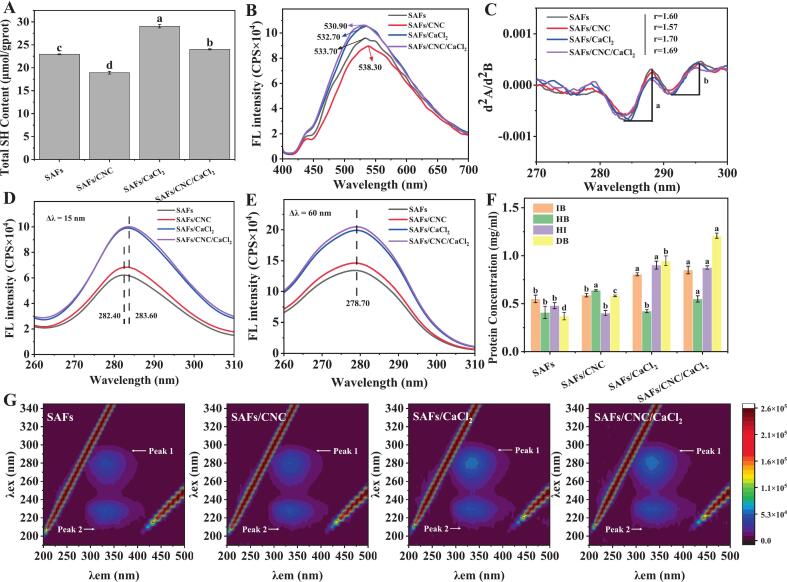
(A) Total sulfhydryl content, (B) surface hydrophobicity, (C) ultraviolet second derivative spectra, (D and E) synchronous fluorescence spectra, (F) intermolecular interactions, and (G) three-dimensional fluorescence spectral contour plot of SAFs-based composite solutions. Different letters (a-d) indicate significant differences between the different treatment groups (*p* < 0.05).

In Fig. 4A, a notable reduction of 17.49 % in the total sulfhydryl content is observed when comparing SAFs/CNC to SAFs. Research has found that a reduction in total sulfhydryl content of the gel indicates further disulfide bond formation (Han et al., 2022). So, we presumed that CNC could have promoted the transformation of sulfhydryl groups of SAFs to disulfide bonds during heating and the oxidation of sulfhydryl groups to form more disulfide bonds, or some sulfhydryl groups were covered up during the cross-linking process of CNC and SAFs. Conversely, the total sulfhydryl content exhibited a significant increase of 26.69 % for SAFs/CaCl_2_ and 4.75 % for SAFs/CNC/CaCl_2_, when compared to SAFs (*p* < 0.05). It was found that the adding of Ca^2+^ could promote the development of protein conformation and raise the content of sulfhydryl group in the conversion process of α-helix to β-folding (Liu et al., 2019).

#### 3.6.2 Surface hydrophobicity

The surface hydrophobicity of the solution after adding CNC declined remarkably compared with SAFs (Fig. 4B). This might be because the cross-linking between SAFs and CNC will consume part of the surface hydrophobic groups of SAFs, thus resulting in the reduction of surface hydrophobicity (Miao et al., 2023). Alternatively, CNC created a space hindrance that overrided the hydrophobic spots of the protein surface, resulting in fluorescence quenching. In addition, the aggregates formed by CNC-induced SAFs, by burying hydrophobic residues, hinder their binding with 8-aniline-1-naphthalene sulfonic acid, ultimately leading to a reduction in surface hydrophobicity. However, compared with SAFs, the addition of Ca^2+^ caused a sharp rise in surface hydrophobicity, which may be that Ca^2+^ was able to affect the charge balance and make Ca^2+^ compete with the protein for water molecules, resulting in an increased tendency of the protein to repel water molecules and aggregate, thereby increasing the surface hydrophobicity of the protein.

#### 3.6.3 Microenvironment of amino acid residue

*(1) Ultraviolet spectrum*

As depicted in Fig. 4C, two peaks with positive values (289 nm and 296 nm) and two valleys with negative values (284 nm and 292 nm) were observed. Among them, the absorption peak at 289 nm is a characteristic manifestation of the interaction between tyrosine and tryptophan, while the ultraviolet absorption peak at 296 nm specifically represents tryptophan. Some studies indicate that the ratio of positive absorption peak to negative absorption peak (r = a/b) in the above UV spectrogram can be used to reflect the change of tyrosine and tryptophan microenvironment (Qiu, Xia, & Jiang, 2014).The binding of SAFs to CNC may result in the masking of some exposed tyrosine residues, which may result in a reduce in the r value of SAFs/CNC. The R-value of SAFs/CaCl_2_ and SAFs/CNC/CaCl_2_ bigger by 6.25 % and 5.63 %, respectively. The introduction of Ca^2+^ may result in alterations in the protein's configuration, causing the tertiary structure of the SAFs protein to unfold.

*(2) Synchronous fluorescence*

Compared with SAFs, the adding of CNC or/and Ca^2+^ enhanced the fluorescence strength of tyrosine and tryptophan to varying degrees, and the adding of Ca^2+^ resulted in a subtle redshift in the fluorescence peak of tyrosine (from 282.40 nm to 283.60 nm) (Fig. 4D-E). CNC or/and Ca^2+^ may cause conformation changes in SAFs proteins, exposing more aromatic amino acid residues and thus affecting the amino acid microenvironment. The generation of redshift may have been caused by the adding of Ca^2+^ bringing a lot of charge to the amino acid microenvironment, which may have affected the hydrogen bond network of water molecules and generated more water molecular interactions, leading to an enhancement in the polarity of the microenvironment.

*(3) Three-dimensional fluorescence*

The results of three-dimensional fluorescence spectrum analysis were shown in Fig. 4G. The aromatic amino acid signature peak (Peak 1) of SAFs enhanced with the addition of CNC or/and Ca^2+^, and both the peak area and the color grade of the absorption peak increased to varying degrees. This further proves that both CNC and Ca^2+^additives may alter the chemical environment around the amino acid residues of SAFs. It was found that certain changes in the backbone of protein polypeptide chains can be inferred by analyzing the Peak area of Peak 2 (Xu et al., 2022). Both the alone and combined adding of CNC and Ca^2+^ efficiently increased the area of the properties peak (Peak 2), and the combined treatment of both showed a synergistic strengthening effect. CNC and/or Ca^2+^ may promote protein cross-linking, causing changes in protein spatial structure and altering the skeleton length of peptide chains.

### 3.7 Molecular interactions

#### 3.7.1 Intermolecular forces

As illustrated in Fig. 4F, the ionic bond content of the gel after the addition of CNC had no significant change compared with that of SAFs gel, the ionic bond content of SAFs/CaCl_2_ and SAFs/CNC/CaCl_2_ gels enhanced by 46.51 % and 54.65 %, respectively (*p* < 0.05). It could be that Ca^2+^ interacts with the protein to form “salt bridges” that creates more ionic bonds. The enhancement of hydrogen bond interaction in gels may have been more closely related to CNC, and the hydrogen bond content in SAFs/CNC and SAFs/CNC/CaCl_2_ gels enhanced by 56.25 % and 34.38 %, respectively (*p* < 0.05). This may have been because the surface of CNC had a lot of hydroxyl groups that could bind to SAFs, thus increasing the quantity of hydrogen bonds in the gel.

Notably, CNC decreased the hydrophobic interaction in the gel, which aligned with the previously obtained results for surface hydrophobicity. This may have been because the addition of CNC generated steric hindrance, covering the hydrophobic binding site of the protein surface. The existence of Ca^2+^ would enhance the extension and reduction of the double helix structure of protein molecules, expose more hydrophobic groups, and thus lead to stronger hydrophobic interactions (Deng et al., 2020). In comparison to SAFs gels, the disulfide bond content in the composite gels increased significantly. This could be attributed to the fact that CNC, being a polysaccharide, absorbed water surrounding the protein, thereby strengthening the interaction between the polysaccharide and the protein. Consequently, this facilitated the conversion of sulfhydryl groups within the protein into disulfide bonds (Zhao et al., 2024). Ca^2+^ could cause a change in the structure of SAFs, leading to the exposure and conversion of sulfhydryl groups to disulfide bonds, thereby increasing the quantity of disulfide bonds in the gel.

#### 3.7.2 Molecular docking

**Fig. 5:**
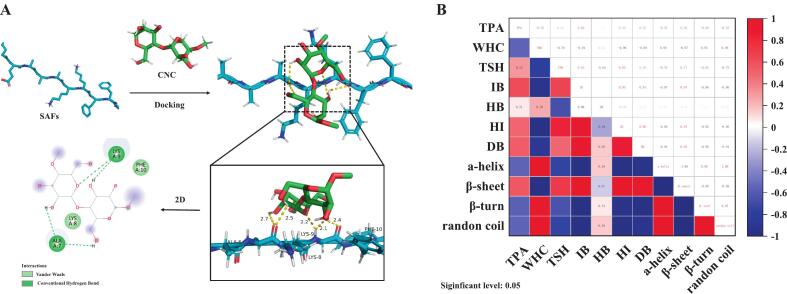
(A) Molecular docking diagram of SAFs and CNC. (B) Pearson correlation analysis of selected factors in the SAFs-based composite gels.

Molecular docking technology is used to explore the intermolecular interactions between receptors and ligands by their characteristics. In Fig. 5A, CNC interacted with two amino acid residues (Phe 10 and Lys 8) on SAFs through van der Waals forces. Moreover, the surface hydroxyl group of CNC was bound to the amino acid residues at Ala 7 and Lys 9 sites on SAFs through hydrogen bond interaction, which also verified the result that the hydrogen bond interaction of the gel increased after the adding of CNC in the intermolecular force. Molecular docking results further proved the existence of the interaction between CNC and SAFs, and CNC was able to associate with specific amino acid residues on SAFs through van der Waals forces and hydrogen bonding. This may be an important reason why CNC can enhance the gel structure.

### 3.8 Correlation analysis

A heatmap of correlations was generated using Pearson correlation analysis to discover the connection between gel strength and WHC of SAFs gel and other indexes after composite treatment. As Fig. 5B showed that there was a remarkable negative relationship between gel intensity and WHC (*r* = −0.55), α-helix (r = −0.55), β-turn angle (*r* = −0.56) and random coiling (r = −0.55), and a remarkable positive relationship (*p* < 0.05) with total sulfhydryl groups (*r* = 0.32), ionic bonds (*r* = 0.60), hydrogen bonds (*r* = 0.079), hydrophobic interactions (*r* = 0.49), disulfide bonds (*r* = 0.52) and β-fold (*r* = 0.56). There was a remarkable negative relationship (*p* < 0.05) between WHC and gel strength (r = −0.55), total sulfhydryl group (*r* = −0.79), ionic bond (*r* = −0.94), hydrophobic interaction (*r* = −0.95), disulfide bond (*r* = −0.89) and β-fold (*r* = −0.97), but a remarkable positive relationship (*p* < 0.05) with hydrogen bond (*r* = 0.24), α-helix (*r* = 0.98), β-turn angle (*r* = 0.95) and random coil (r = 0.98). For gel strength, the three indexes with the largest R-value, namely ionic bond, disulfide bond, and β-fold, were the main factors affecting the intensity of the protein gel. For WHC, the three indexes with the largest R-value: α-helix, β-angle, and random crimp were the major elements affecting the WHC performance of the protein gel.

### 3.9 Comprehensive discussion

**Fig. 6:**
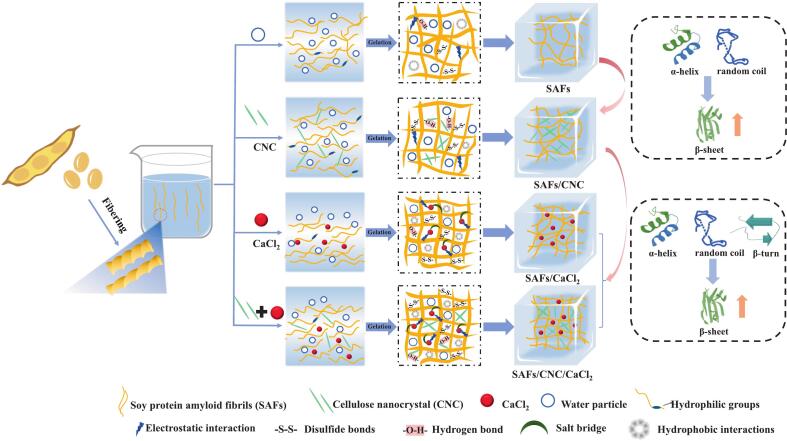
Schematic diagram of the regulatory mechanism of CNC and Ca^2+^ on the performance of SAFs-based composite gels.

Based on the four aspects of protein aggregation behavior, multi-scale structure, protein functional groups and intermolecular force, we investigated the role of CNC and Ca^2+^ in the regulation of the gel properties of SAFs (Fig. 6). Appropriate addition of CNC increased the energy storage modulus, loss modulus, viscosity, gel intensity, and WHC of the SAFs gel. 1.) From the viewpoint of protein aggregation behavior, CNC had no function on the aggregation of SAFs molecules, and the turbidity and solubility showed no significant changes. 2.) Considering the multi-scale structural viewpoint, CNC was conducive to the conversion of α-helix to β-folding, which improved the thermal gel formation ability of proteins. In addition, CNC could enhance the crosslinking between SAFs, reduce the microporous in the gel structure, and thus enhance the “trapping” of water molecules to improve WHC of the gel. 3.) From the viewpoint of the functional groups of proteins, CNC decreased the total sulfhydryl level and surface hydrophobicity of SAFs, while increasing the content of aromatic amino acid residues. 4.) Intermolecular forces Results showed that CNC enhanced the intermolecular forces such as disulfide bond and hydrogen bond of SAFs gel.

The addition of Ca^2+^ increased the storage modulus, loss modulus, viscosity, and gel intensity of the SAFs gel, but decreased its WHC. 1.) From the perspective of protein aggregation behavior, Ca^2+^ caused electrostatic shielding, creating ‘salt bridges’ that caused protein aggregation, which resulted in an growth in turbidity and a decline in solubility of SAFs solutions. 2.) From the perspective of multi-scale structure, it was found that the addition of Ca^2+^ was conducive to the transformation of α-helix, β-corner and irregular curling into β-folding, and the growth of β-folding was conducive to the enhancement of thermal gel formation capacity. Ca^2+^ also induced conformational rearrangement of SAFs molecules, leading to the formation of new amorphous conformations with lower crystallinity. In addition, Ca^2+^ could make the protein in the SAFs gel more tightly cross-linked, and the lamellar structure became thicker, the surface denser but with larger gaps. Such changes enhanced the SAFs gel's gel strength but also reduced the WHC of the gel. 3.) From the functional groups of proteins, Ca^2+^ rose the total sulfhydryl quantity and surface hydrophobicity of the gel, and exposed more aromatic amino acid residues. 4.) The change in protein conformation exposed more sulfhydryl groups, which helped to form more disulfide bonds and improved the gelation properties of the gel. In addition, Ca^2+^ also enhances ionic bonding and hydrophobic interactions of protein gels, leading to the development of larger protein aggregates.

The compound gel containing CNC and Ca^2+^ had the highest energy storage modulus, loss modulus, viscosity, and gel strength, which may have been the synergistic effect of the two on the performance of the SAFs gel. Comparing the difference between CNC and Ca^2+^, it was found that the two strengtheners showed increased characteristics in β-folding, thermal stability, aromatic amino acid content and disulfide bond, which may be a significant reason for the maximum gel performance of SAFs/CNC/CaCl_2_ gel. In addition, Ca^2+^ increased the above indexes to a greater extent than CNC, indicating that Ca^2+^ played a major role in the SAFs/CNC/CaCl_2_ system. The difference was that CNC led to the conversion of α-helix and β-corner to β-fold in the SAFs gel, while the presence of Ca^2+^ not only led to the conversion of α-helix and β-corner to β-fold, but also increased the amount of β-fold in the gel by converting additional random coils to β-fold.

In general, both CNC and Ca^2+^ could realize the control of the performance of SAFs gels through the transformation of multi-scale structure, the exposure of protein functional groups (such as aromatic amino acids), and the enhancement of related intermolecular forces. In addition, Ca^2+^ could also promote cross-linking of protein, thereby enhancing the protein aggregation. Together, these factors improved the gel performance of SAFs, especially when they were treated together.

## 4 Conclusion

The gel characteristics of SAFs could be regulated by the appropriate amount of CNC (0.09 %, *w*/*v*) and CaCl_2_ (400 mmol) and the synergies between the two. Both single and collaborative treatments of CNC and Ca^2+^ promoted the formation of β-sheet conformations and the changes in amino acid environment. CNC promoted the formation of “thin-layer” gel structural units in SAFs with superior WHC and high gel strength. However, Ca^2+^ promoted the formation of “thick slices” gel structural units in SAF molecules with low WHC and high gel strength. The findings indicated that CNC and Ca^2+^ could achieve diversified regulation of SAFs gel properties by varying the aggregation behavior, multi-scale structure, functional group distribution, and intermolecular interactions of SAFs molecules. Future research could focus on expanding the application potential of CNC/Ca^2+^ regulated protein fibril gels in the encapsulation and delivery of bioactive substances, as well as the design of gel structures for functional foods with specific texture and water retention properties. This had important reference value for the exploitation of SAFs gel-based goods.

## CRediT authorship contribution statement

**Shanlong Zhu:** Writing – original draft. **Xinyuan Song:** Methodology. **Kang Zhong:** Methodology. **Lu Lin:** Software. **Ye Huang:** Visualization. **Wenbin Zha:** Formal analysis. **Yingnan Liu:** Validation. **Wei Lan:** Validation. **Yaqing Xiao:** Writing – review & editing.

## Declaration of competing interest

The authors declare that they have no known competing financial interests or personal relationships that could have appeared to influence the work reported in this paper.

## Data Availability

Data will be made available on request.

## Appendix A Supplementary data

Supplementary material Image 1
