# Supplementary material for: Improvement mechanism of cellulose nanocrystal in coordination with calcium ions on the thermal gelation of soybean protein amyloid fibrils
Source: Food Chem X. 2025 Nov 7;32:103275. doi: 10.1016/j.fochx.2025.103275 (PMC12663481; doi:10.1016/j.fochx.2025.103275)
Supplement: Supplementary file 1 — Supplementary material [file mmc1.docx]

**Improvement mechanism of cellulose nanocrystal in coordination with calcium ions on the thermal gelation of soybean protein amyloid fibrils**

Shanlong Zhu^a,b^, Xinyuan Song^b^, Kang Zhong^b^, Lu Lin^b^, Ye Huang^b^, Wenbin Zha^b^, Yingnan Liu^b^, Wei Lan Wei Lan^a,^*, Yaqing Xiao^b,^*

*a Anhui Ecological Fermentation Engineering Research Center for Functional Fruit Beverage, Fuyang Normal University, Fuyang, 236037*

*b Key Laboratory of Jianghuai Agricultural Product Fine Processing and Resource Utilization of Ministry of Agriculture and Rural Affairs, College of Food and Nutrition, Anhui Agricultural University, Hefei, 230036, China*

*Wei Lan and Yaqing Xiao are the corresponding author

E-mail addresses: lanwei@fynu.edu.cn; xiaoyaqing92@163.com

**
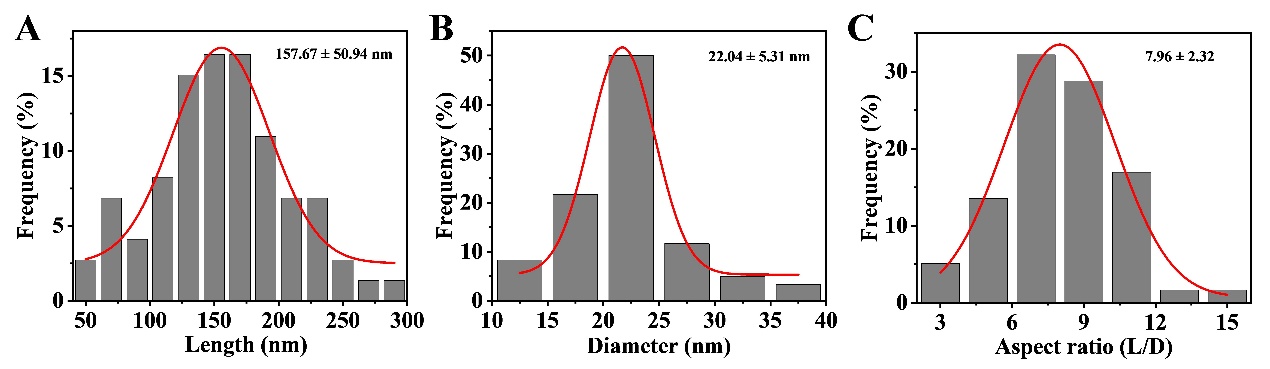
Fig. S1.** (A) Length distribution histogram, (B) diameter distribution histogram, and (C) aspect ratio distribution histogram of the CNC prepared from pear peel pomace. CNC: cellulose nanocrystal.


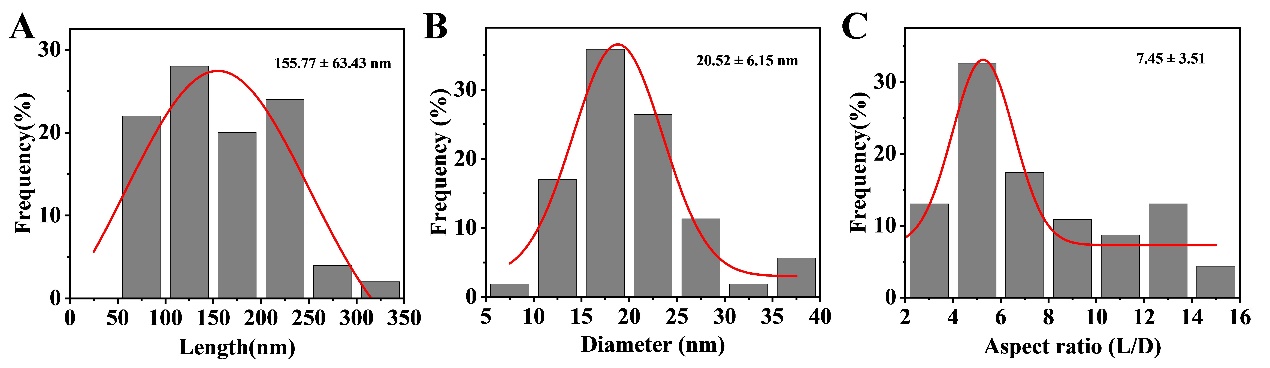


**Fig. S2.** (A) Length distribution histogram, (B) diameter distribution histogram, and (C) aspect ratio distribution histogram of SAFs. SAFs: soybean protein amyloid fibrils.
